# Influence of Oil Polarity and Cosurfactants on the Foamability of Mono- and Diacylphosphatidylcholine Stabilized Emulsions

**DOI:** 10.3390/pharmaceutics14061212

**Published:** 2022-06-07

**Authors:** Manuel Bunk, Rolf Daniels

**Affiliations:** Department of Pharmaceutical Technology, Eberhard Karls University, Auf der Morgenstelle 8, 72076 Tuebingen, Germany; manuel.bunk@uni-tuebingen.de

**Keywords:** phospholipids, monoacylphosphatidylcholine, diacylphosphatidylcholine, foam, emulsion, profile analysis tensiometry (PAT), laser diffraction measurements

## Abstract

Foam formulations are safe and effective therapy options for the treatment of chronic skin conditions that require the application of a topical formulation to delicate skin areas, such as scalp psoriasis or seborrheic dermatitis. This study focused on the development of foamable emulsions based on aqueous phospholipid blends. The effects of cosurfactants (nonionic Lauryglucoside (LG); zwitterionic Lauramidopropyl betaine (LAPB)), as well as of oil phases of different polarities, namely paraffin oil (PO), medium-chain triglycerides (MCT) and castor oil (CO), were investigated. The foaming experiments showed that both the type of cosurfactant, as well as the type of oil phase, affects the quality of the resulting foam. Emulsions that were based on a combination of hydrogenated lysophosphatidylcholine (hLPC) and a non-hydrogenated phospholipid, as well as LG as a cosurfactant and MCT as an oil phase, yielded the most satisfactory results. Furthermore, profile analysis tensiometry (PAT), polarization microscopy and laser diffraction analysis were used to characterize the developed formulations. These experiments suggest that the employed phospholipids predominantly stabilize the emulsions, while the cosurfactants are mainly responsible for the formation and stabilization of the foams. However, it appears that both sets of excipients are needed in order to acquire stable emulsions with satisfactory foaming properties.

## 1. Introduction

Among the most common reasons for poor compliance in the treatment of chronic skin conditions is the choice of subpar vehicles. As a result, this often leads to reduced therapy success [1]. Contrary to more conventional dosage forms such as creams and ointments, foam formulations distinguish themselves in high consumer acceptance and consequently improved patient compliance [2]. Foam formulations can be applied with negligible mechanical stress [3], which is especially important when treating irritated or sensitive skin. Other advantages include the ease of application to hair-bearing skin [4], as well as the improved skin penetration and bioavailability of drugs [5]. Moreover, foaming allows to transform a liquid into a spreadable formulation for easier application to, e.g., the legs. In recent years, foam formulations have established themselves as important therapy options for a multitude of indications, such as androgenic alopecia [6] and even non-dermatological conditions such as ulcerative colitis [7] or vaginal infections [8].

Phospholipids (PL) have also been of tremendous interest in topical drug delivery and skin applications for a long time now [9]. As a part of cellular membranes, they exhibit a high degree of skin compatibility [10] and have been deemed safe for use in cosmetic applications by the Cosmetic Ingredient Review (CIR) organization [11,12].

Unique characteristics like the ability to form various different aggregates in an aqueous environment, such as lamellar vesicles (e.g., liposomes) and micelles, result in interesting interfacial properties for emulsion stabilization and foam formation [13]. This, combined with their overall technological versatility and modifiability, makes phospholipids perfect candidates for the development of foamable formulations [14].

**Figure 1:**
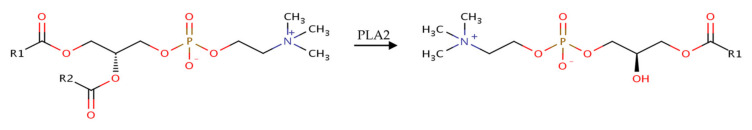
Biochemical modification of native phosphatidylcholine (PC).

Lysophosphatidylcholine (LPC) in particular is a promising excipient in this context due to its surfactant-like behavior and good aqueous solubility [15]. Compounds with increased amounts of LPC can generally be acquired by chemically modifying phospholipids from natural sources such as soybeans or egg yolks with enzymes of the phospholipase family. Phospholipase A2 (PLA2) cleaves the fatty acid from the sn-2 position [16], as illustrated in Figure 1.

Interestingly, there is almost no research or literature published specifically on foam stabilization using lysophosphatidylcholine. To the best of our knowledge, the only published article in this context focused on the thickness measurement of foam films with lysophosphatidylcholine [17].

To fill this gap, the aim of this study was to analyze various LPC-containing phospholipid blends in regard to their foaming abilities. Moreover, emulsions based on these phospholipid premixes were prepared to study the effects of different oil phases and cosurfactants. To further the understanding of the foaming behavior of such formulations and the differences in foam and emulsion stabilization, pendant drop tensiometry, as well as polarization microscopy, were used.

## 2. Materials and Methods

### 2.1. Materials

Plantacare^®^ 1200 UP and Kollisolv^®^ MCT 70 (BASF SE, Ludwigshafen am Rhein, Germany), TEGO^®^ Betain F 50 (Evonik Nutrition & Care GmbH, Essen, Germany), White soft paraffin and refined castor oil (Caesar & Loretz, Hilden, Germany) were used.

Hydrogenated phospholipids from soybeans with 20% lysophosphatidylcholine (Lipoid S LPC 20-3) (LPC20), as well as Lipoid P LPC 90 (LPC90), Phospholipon^®^ 80 H (PL80H) and Phospholipon^®^ 90 G (PL90G), were kindly provided by Lipoid GmbH, Ludwigshafen am Rhein, Germany. The chemical compositions of the phospholipid compounds are summarized in Table 1.

### 2.2. Preparation of Premixes

The aqueous phospholipid premixes used in this work consisted of a blend of two phospholipid compounds as the dispersed phase.

In those premixes containing LPC90, this substance was dissolved in purified water first, using a magnetic stirrer (Heidolph Instruments GmbH & CO. KG, Schwabach, Germany) at 350 rpm. Thereafter, the remaining phospholipid components were added, and the mixture was homogenized at 45 °C using a propeller stirrer (Heidolph Instruments GmbH & CO. KG, Schwabach, Germany) at 1400 rpm for 15 min. All premixes were analyzed 24 h after preparation.

### 2.3. Preparation of Oil-in-Water Emulsions

Three aqueous phospholipid premixes were chosen for the emulsion development. In total, 12 emulsions were prepared that consisted of a phospholipid premix and an oil phase (white soft paraffin (PO), medium-chain triglycerides (MCT) and refined castor oil (CO)), as well as a cosurfactant. The cosurfactants used were Plantacare^®^ 1200 UP (main component: Lauryl glucoside (LG)) and TEGO^®^ Betain F 50 (main component: Lauramidopropyl betaine (LAPB)).

During the first emulsification step, the respective oil was added to the aqueous phospholipid premix and homogenized for 4 min at 9000 rpm and 2 min at 13,400 rpm using an Ultra Turrax T 25 disperser (IKA-Werke GmbH & Co. KG, Staufen, Germany) equipped with a S 25 N-18 G dispersing tool. After adding the cosurfactant, the preparation of the emulsions was completed using an ultrasonic homogenizer (UP200S ultrasonic processor, Hielscher Ultrasonics GmbH, Teltow, Germany) equipped with a S7 titanium sonotrode with an amplitude setting of 50% (0.5 cycle) for 5 min. All emulsions were analyzed 24 h after preparation.

### 2.4. Foam Analysis

**Figure 2:**
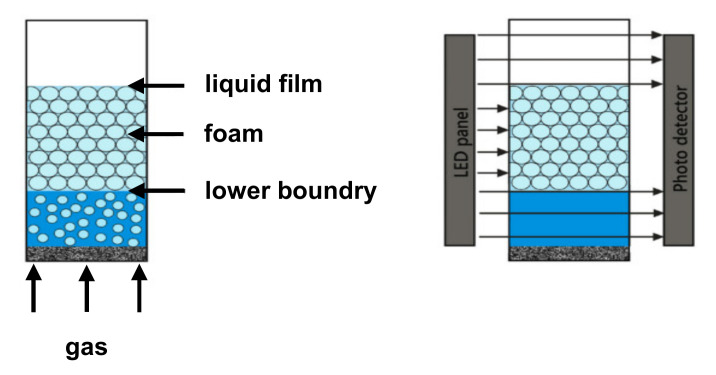
Foaming and height detection by means of the DFA100.

Foaming and foam characterization were performed using a Dynamic Foam Analyzer—DFA100 (Kruess GmbH, Hamburg, Germany), equipped with a foam structure module (FSM). Figure 2 depicts the foaming principle and the foam height detection of the DFA100.

The foam height is measured via a light-emitting diode (LED) panel and a photodetector while the glass column with the foam is located in between. The light emitted by the LED panel is detected at the line sensor after passing through the glass column. Since liquids and gas are transparent and foam is less transparent, the liquid/foam and foam/gas boundaries can be detected in this way.

**Figure 3:**
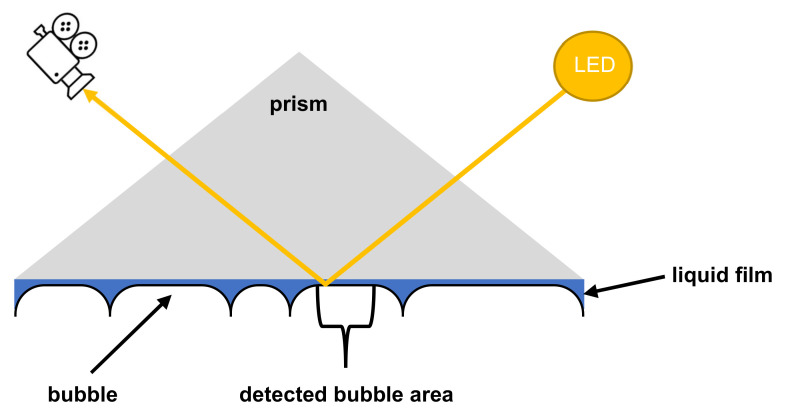
Scheme of the measuring principle of the structure detection applied by the DFA100.

The FSM consists of an optical sensor, a secondary illumination source (structure illumination) and a glass column equipped with a prism to allow two-dimensional imaging of the foam structure without the curvature of the glass column. Figure 3 shows how air bubbles cause total reflection of the light from the structure illumination and therefore cause the optical sensor to register an image. The mean bubble area (MBA) (µm^2^) and the bubble count (BC) (1/mm^2^) were determined as a measure of the foam structure. The calculation of these parameters is done automatically by the accompanying software ADVANCE v. 1.6.2.0.

Air was utilized as a propellant using a flow rate of 0.4 L/min. The quality of the foam was evaluated by its foamability (initial foam height) and foam stability expressed as the foam height at three individual time points (t_0_, t_150_ and t_300_) over the course of a total measuring time of 360 s.

### 2.5. Droplet Size Analysis

A Mastersizer^®^ 2000 (Malvern Instruments Ltd., Malvern, UK) with a Hydro 2000S module was used to characterize the cumulative volume-based droplet size distribution of the emulsion samples by means of the laser diffraction technique. The experiments were conducted at room temperature, and the results are expressed as the d_10_, d_50_ and d_90_ values.

About 30 µL of the emulsion sample was diluted in purified water under stirring (1750 rpm) in the Hydro 2000S module until an obscuration of 2–6% was reached. For each emulsion, three aliquots were measured with each measurement cycle consisting of three separate recordings of a 30-s period.

### 2.6. Density Measurements

The required densities of the samples for the subsequent tensiometry measurements were determined at a temperature of 25 °C by means of a DMA 4500 density meter (Anton Paar, Graz, Austria) based on the oscillating U-tube technique. In order to prevent air bubbles in the tube, the samples were inserted carefully after a cleaning and drying process was performed every time a new sample was characterized. The determination was carried out in triplicate.

### 2.7. Surface Tension Measurements

Pendant drop tensiometry was used to determine the surface tension (liquid/air interface) of the samples. For each sample, five individual measurements were conducted, and purified water was measured as a reference.

The experiments were performed at a temperature of 25 °C and included 900 measuring points over the course of 15 min. The surface tension was calculated from fitting the drop shape to the Young-Laplace equation [18].

The transport of surface active ingredients in liquid formulations is known to be a diffusion process, which is inversely proportional to the square root of time, and the surface tension decreases over the course of a measurement before reaching a steady level [19]. Therefore, values from 600 s to 900 s were used to determine the equilibrium surface tension of each sample.

The measurements were performed on a PAT-1D (Sinterface Technologies e.K., Berlin, Germany), and the profile of the drop was fitted to the Young-Laplace equation using the accompanying software PAT 1 v. 5.03.1 (Sinterface Technologies e.K., Berlin, Germany). The fitted data was then plotted as σ (1/√t), and the surface tension was determined as the y-intercept of the extrapolation of the measured data [20,21].

### 2.8. Microscopy

Foam samples were applied thinly to a microscope slide and covered carefully with a coverslip. The foam samples were generated through mild manual shacking of the formulation immediately prior to the application.

An Axio Imager Z1 microscope (Carl Zeiss, Jena, Germany) with crossed polarizers and a λ/4-plate was used to capture the polarization microscopic images at a magnification of 40-fold. The images were taken with a Axiocam 105 camera and processed with Axiovision v. 4.6.3 software.

### 2.9. Statistical Analysis

Whenever it was possible, the results were presented as the mean value of the observed parameter ± SD. Statistical analysis was conducted with the help of GraphPad Prism 8.0 (GraphPad Software Inc., San Diego, CA, USA).

Statistical differences among multiple groups were evaluated through one-way ANOVA (analysis of variance), followed by Tukey’s multiple comparisons test. Assessment of the statistical significance in the foaming values generated from formulations with and without a cosurfactant was performed through a Student’s *t*-test. Significant differences were marked with a number of asterisks (*) as follows: * *p* ≤ 0.05, ** *p* ≤ 0.01, *** *p* ≤ 0.001 and **** *p* ≤ 0.0001. Only the differences related to the discussion are marked with asterisks in the graphs for better clarity.

## 3. Results and Discussion

### 3.1. Influence of Phospholipid Premix Composition on Foaming Properties

All manufactured premixes were turbid liquid dispersions with a water-like consistency. Blends with varying concentrations (0.25%, 0.50% and 1.00%), as well as different ratios of PL1:PL2 (3:1, 1:1 and 1:3), of two phospholipids were prepared. The most suitable total phospholipid concentration and PL1:PL2 ratio for each premix has been determined in a previous study (data not shown). Table 2 shows a summary of the investigated premixes.

**Figure 4:**
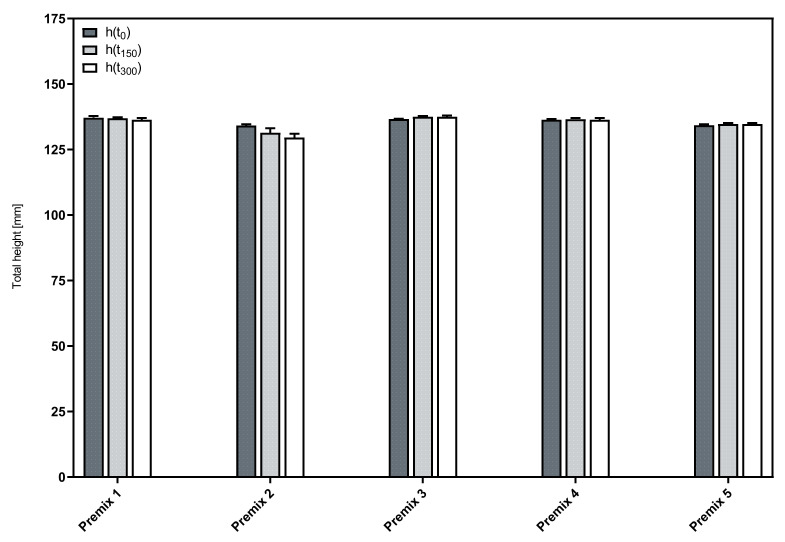
Time course of the total foam height of the aqueous phospholipid premixes; mean ± SD, *n* = 3.

Figure 4 shows the total height of the foamed premixes and its change over time. The negligible differences in the initial foam height (h(t_0_)) for all formulations indicate that the foamability is neither influenced by the composition nor the concentration of the phospholipid blend used as a foaming agent.

Most premixes show a minute increase in height during the measuring time. This observation can potentially be explained by an expansion of the individual bubbles due to the internal pressure acting against a decreasing strength of the foam bubble membranes as a result of liquid drainage [22]. In combination with limited decay, this could lead to a slight increase in the total volume over the course of a measuring time as short as 5 min. The only exception from this general behavior was observed with Premix 2, which was the only premix that contained predominantly LPC90 (75% of the phospholipid blend). It showed a slight decrease in total height over the course of the measurements. Obviously, here, the decay was marginally larger than the post-expansion of the compressed air after the foaming process. However, in summary, all premixes allow to form stable foams.

**Figure 5:**
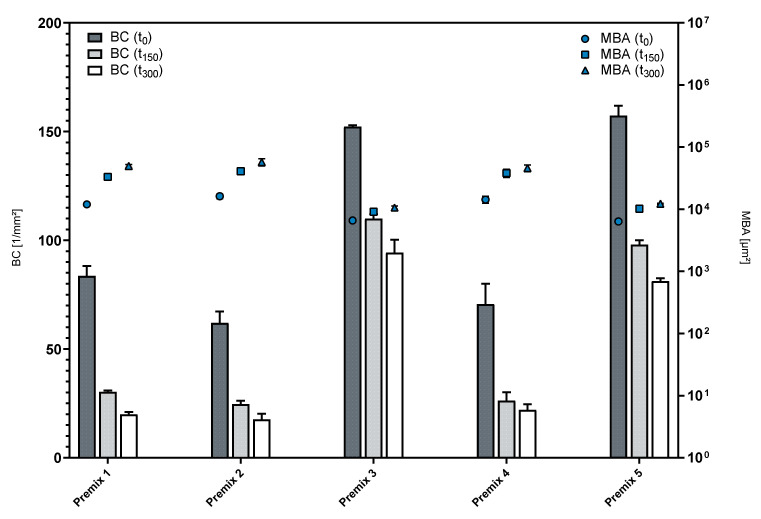
Structure of foamed premixes over time; mean ± SD, *n* = 3.

The foam structure analysis (FSA) is used to determine the structural attributes of foamed formulations and their progression over time. It is conducted with the foam structure module (FSM) of the DFA100, which includes a secondary illumination source, as well as a high-resolution camera and a modified glass column with an attached prism [23]. Foam structure is expressed as the bubble count per mm^2^ (BC) and the mean bubble area (MBA). Figure 5 depicts these parameters for the foamed premixes over the course of a measurement.

Premix 3 and Premix 5 yielded highly dispersed foams, as indicated by the high bubble count and relatively small mean bubble area in comparison to the other formulations. Both formulations also showed superior foam stability, as changes in either parameter over time were less pronounced than in the rest of the examined formulations, as displayed in Table 3. While Premixes 3 and 5 were able to maintain 61.93% and 51.69% of their initial bubble count, respectively, the foam structure parameters of the other premixes showed substantial decay. This was most pronounced with Premix 1 maintaining only 23.90% of its initial bubble count after 300 s.

The addition of PL80H, a hydrogenated PL, to either of the LPC compounds (Premix 2 and Premix 4) impaired their foamability, becoming obvious by the lower initial BC and higher initial MBA. Additionally, Premix 1 consisting of phosphatidylcholine (PL90G) and lysophosphatidylcholine (LPC90) displayed relatively low foam dispersity and foam structure stability. Therefore, it can be concluded that blends that include predominantly LPC20 with an addition of a non-hydrogenated secondary phospholipid (LPC90 or PL90G) lead to more disperse foams that can maintain their foam structure better than other premixes. The presence of hydrogenated lysophosphatidylcholine (hLPC) appears to play an important role in producing these results, as neither of the premixes that only included non-hydrogenated lysophosphatidylcholine (LPC) (Premixes 1 and 2) displayed similarly high foam dispersity or stability. This observation is in line with the increased elasticity of LPC-containing phospholipid membranes [24], since film elasticity plays a vital role in the stabilization of foams [25]. Taking all of the previously mentioned observations into account, Premixes 3 and 5 are the most suitable candidates for the development of a foamable phospholipid emulsion.

### 3.2. Foaming Behavior of Phospholipid Emulsions

Based on the findings in Section 3.1, the further experiments dealing with the foamability of emulsions were based on Premixes 3 and 5.

Premix 3 was not sufficient to simultaneously stabilize the emulsion and the foam. In the case of Premix 5, the overall foam quality of the resulting emulsion was disappointing compared to the pure premix investigated previously. Therefore, the use of a cosurfactant was considered. For more detailed information, the reader is kindly referred to Supplementary Materials Figures S1–S7.

**Figure 6:**
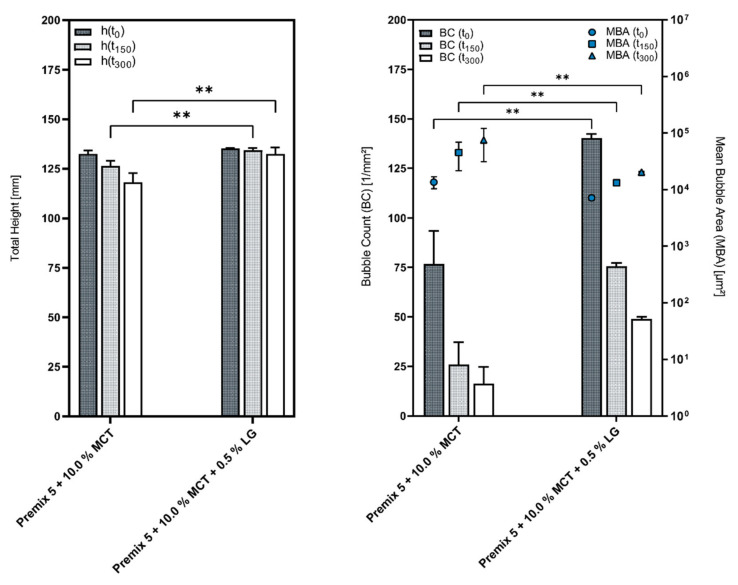
Comparison of the foams generated from Premix 5 + 10.0% MCT with and without a cosurfactant; mean ± SD, *n* = 3; ** *p* ≤ 0.01.

Supplementing these emulsions with a cosurfactant enhances the foam quality, both in regard to the height stability, as well as the foam structure, and, additionally, provides more consistent results overall, as illustrated by Figure 6.

### 3.3. Influence of the Cosurfactants and Polarity of the Oil Phase on the Foaming Properties of Phospholipid Emulsions

The results of Section 3.2 show that the incorporation of an additional surface-active component is beneficial in order to obtain stable, foamable emulsions based on phospholipid Premixes 3 and 5 discussed in Section 3.1. Further systematic experiments use these two premixes and three different oil phases representing a broad range of polarities, namely paraffin oil (PO), medium-chain triglycerides (MCT) and castor oil (CO). The polarity of these oil phases increases in the order: paraffin oil < medium-chain triglycerides < castor oil. Moreover, the emulsions were supplemented with two classical foam surfactants (nonionic Lauryglucoside (LG); zwitterionic Lauramidopropyl betaine (LAPB)). Table 4 lists all the investigated emulsions.

**Figure 7:**
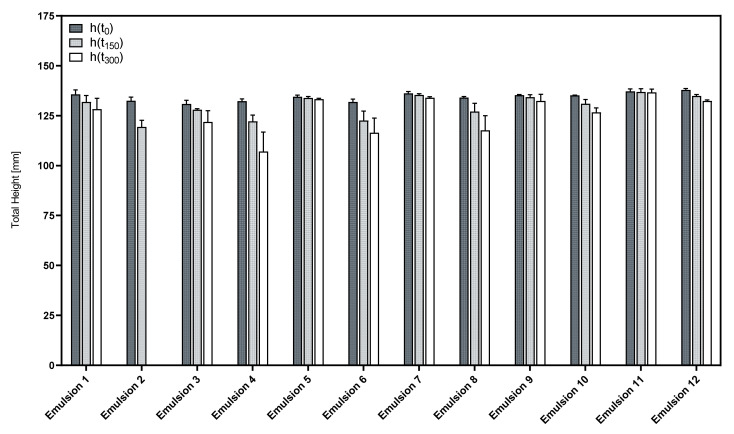
Time course of the total foam height of the phospholipid emulsions; mean ± SD, *n* = 3.

Figure 7 shows the total height of the foamed formulations and its change over time.

The initial foamability of the formulations was neither influenced by the presence of an oil phase nor the use of a cosurfactant, as indicated by the parameter h(t_0_) that is almost identical in all investigated emulsions and premixes.

However, in contrast to the aqueous phospholipid premixes, some of the corresponding emulsions display a much lower foam stability. This could have been expected, as the presence of oily substances is known to impede the formation of stable foam structures [26]. This was especially true for emulsions that included LAPB as a zwitterionic cosurfactant, as indicated by Emulsions 4, 6 and 8. Even worse was the stability of the foam from Emulsion 2, where the foam almost completely decayed after 300 s.

On the other hand, the corresponding formulation variants using LG as a nonionic cosurfactant showed far superior foam stability in comparison. Emulsions 5, 7, 9 and 11 in particular displayed good foam stability close to the level of the pure phospholipid premixes.

**Figure 8:**
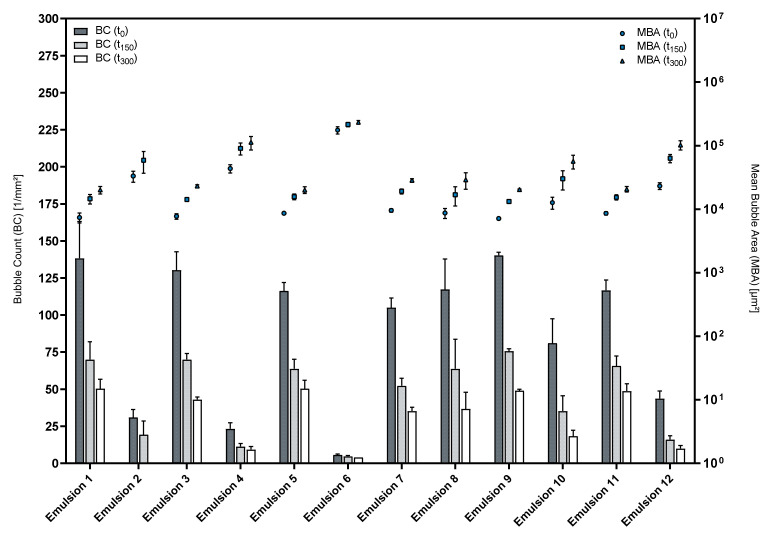
Structures of foamed emulsions over time; mean ± SD, *n* = 3.

Figure 8 shows the respective results for the foam structure analysis. Obviously, the used phospholipid premix largely affects the foam structure. Interestingly, the formulations that were based on Premix 3 (Emulsions 1–6) yielded highly disperse foams whenever LG was used as a cosurfactant, and in contrast, foam formation was almost impossible when LAPB was used as a cosurfactant. In contrast, Premix 5-based emulsions were not influenced by the type of cosurfactant in a similar way.

Emulsions 1, 3 and 9 performed best with respect to the foam structure with Emulsion 9 showing the most reproducible results. Table 5 summarizes the results of the foam structure analysis for these emulsions and the corresponding premixes.

This data shows that the differences in foam structures between the emulsions and their respective premix formulations become more pronounced over the course of a measurement, indicating that the presence of an oil phase influences the foam stability more severely than the initial foam structure.

PO emulsions (1, 2, 7 and 8) displayed similar results to the respective MCT emulsion (3, 4, 9 and 10), with the latter being the more reproducible choice. CO emulsions (5 and 11) using LG as a cosurfactant generally were capable of producing foams of good overall quality in regard to their stability and structural attributes. This indicates that the selection of a medium- to high-polarity oil phase was beneficial for the foam quality of these formulations. However, macroscopically, the CO emulsions were far less stable than most of the PO and MCT emulsions and showed creaming within hours after the preparations. The droplet size distribution of an emulsion is directly related to its creaming rate.

Table 6 shows the results of the droplet size measurements of the tested emulsions.

None of the investigated CO approaches (Emulsions 5, 6, 11 and 12) showed sufficiently small droplets (mean droplet size: 20.165 ± 3.900 µm) for the formulation to be considered stable.

On the other hand, approaches that employed low-polarity PO (Emulsions 1, 2, 7 and 8) and medium-polarity MCT (Emulsions 3, 4, 9 and 10) yielded satisfying results. With a mean droplet size of 3.042 ± 0.344 µm across all formulations, the PO emulsions showed higher values than the MCT emulsions, with a mean droplet size of 2.055 ± 0.223 µm. The same observations applied for the d_10_ and d_90_ values.

The respective LAPB and LG approaches of each premix/oil combination displayed very similar results. It can therefore be assumed that the type of cosurfactant does not influence the droplet size distribution and, therefore, the stability of the emulsions in a similar way as the type and the polarity of the oil phase does.

**Figure 9:**
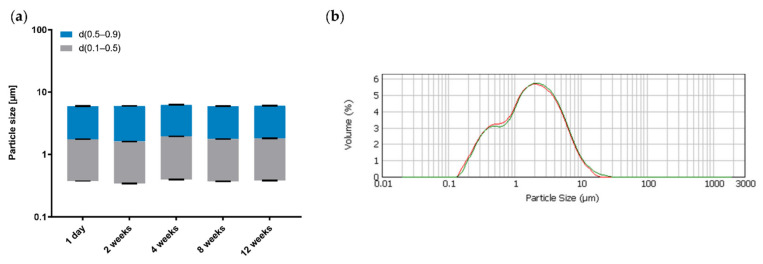
(**a**) The d_10_, d_50_ and d_90_ values during storage. (**b**) Volume-based droplet size distribution on 1 day (red) and 12 weeks (green) after preparation; mean ± SD, *n* = 3.

Figure 9 shows the droplet size distribution of Emulsion 9 over a storage period of 12 weeks at 25 °C in the dark.

During this period, the droplet sizes are unaffected, as indicated by the near-constant results for the d_10_, d_50_ and d_90_ values and the overall volume-based droplet size distribution. It can therefore be assumed that the investigated emulsions consisting of Premix 5, LG and MCT remain physically stable for a period of at least three months when stored at room temperature.

#### Characterization of Liquid–Air Interface 

According to Gibbs Equation (1), a system with a higher dɣ (lower surface tension compared to water) displays a higher concentration of adsorbed surface-active molecules in the adsorption layer, thus indicating higher activity of the foaming agent(s) at the liquid–air interface [1,27].

(1) $$\Gamma =-\frac{c}{RT}*\frac{dɣ}{dc}$$

Γ—Surface concentration of a foaming agent (mol/m^2^);
ɣ—Surface tension of the liquid (N/m);
*c*—molar concentration of the foaming agent (mol/L);
*R*—gas constant (J/mol*K);
*T*—temperature (K).

The liquid–air interface is of particular interest when dealing with foam generation and foam stability [28]. Surface tension and its variations during formulation optimization can give the first hints on the suitability of a formulation approach [29].

To further study the roles of the various surface-active ingredients that are part of the investigated premix and emulsion formulations, surface tension experiments by means of a drop profile analysis using the pendant drop technique were conducted.

In order to acquire comparable results, Premixes 3 and 5 and the corresponding emulsion approaches (Emulsions 7, 9, 10 and 11), as well as aqueous LG (0.5% (*w*/*w*)) and LAPB (0.5% (*w*/*w*)) solutions, were included in these experiments.

**Figure 10:**
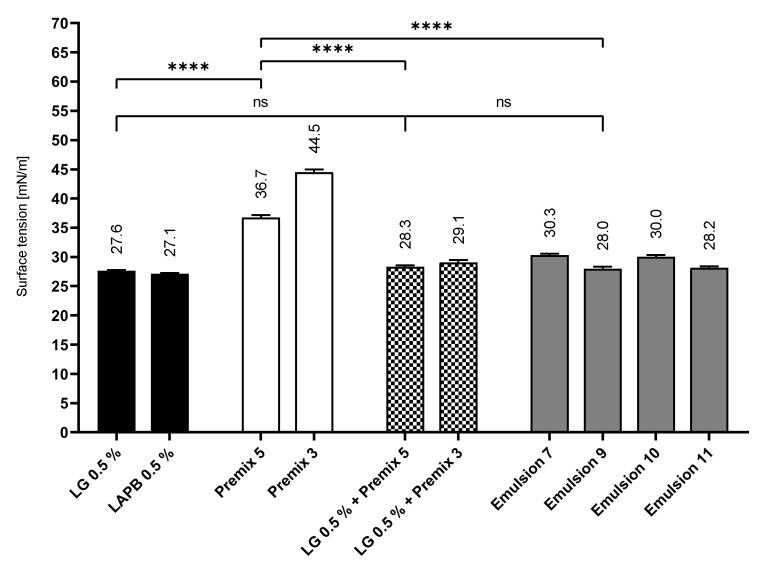
Surface tension of the PL emulsions and precursors; mean ± SD, *n* = 5; **** *p* ≤ 0.0001.

Figure 10 depicts the extrapolated equilibrium surface tension of these samples. Generally, the pure aqueous cosurfactant solutions showed the lowest surface tensions of the investigated samples, while the PL premixes showed the highest values. The surface tension for the premix/cosurfactant mixtures was almost identical to the surface tension of the corresponding MCT and CO emulsions and by far closer to the cosurfactant solution than to the PL premix. Premix 5, for example, showed a high surface tension value of 36.7 mN/m, while the LG solution lowered the surface tension to 27.6 mN/m. The mixture of both yielded a surface tension of 28.3 mN/m, which was much closer to the pure LG solution. Obviously, LG with its more pronounced interfacial activity dominated the behavior of the surfactant/phospholipid blend. This suggests a preferred presence of LG at the liquid–air interface, leading to enhanced foaming properties of the formulations.

**Figure 11:**
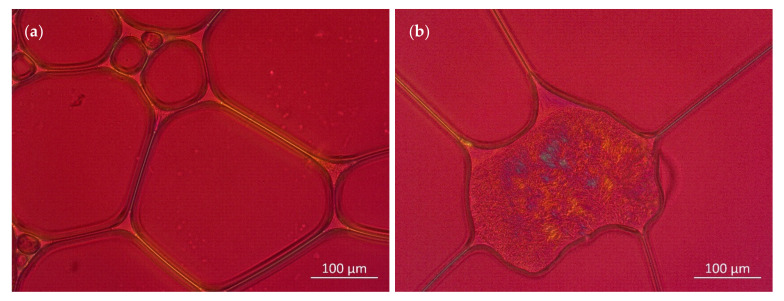
Polarization microscopic images of foams generated from the (**a**) LG + Premix 3 and (**b**) LG + Premix 5.

This assumption is underlined by Figure 11, which shows polarized light microscopic images of foams generated from the LG/Premix 3 and LG/Premix 5 blends, indicating the presence of liquid–crystalline LG structures at the plateau border region and foam lamellae.

Consequently, the phospholipids are likely to be associated with the stabilization of the emulsion. This is also in accordance with the vastly different foaming behaviors of the LG and LAPB approaches discussed in Section 3.3.

Emulsion 7 (28.2 mN/m) and Emulsion 9 (28.0 mN/m) displayed the lowest surface tension among the emulsions, with the respective PO (30.3 mN/m) and LAPB (30.0mN/m) approaches showing noticeably higher values. This could partially explain the more favorable foaming properties of Emulsion 9 when directly compared to Emulsions 7 and 10 because of the tendency of systems with lower surface tensions to produce more stable foams, according to Gibbs equation.

**Figure 12:**
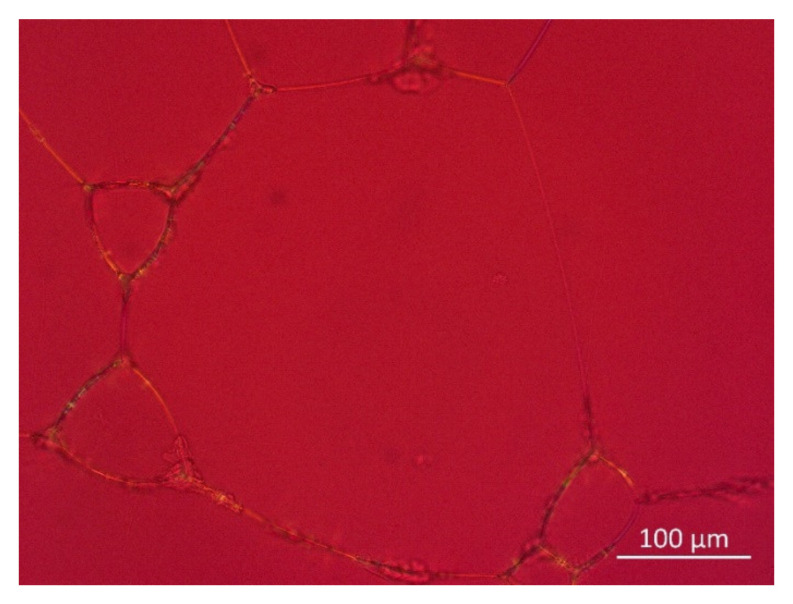
Polarization microscope image of dried-up foam generated from Premix 5.

The high stability of the foams generated from premixes regardless of the higher surface tension can be explained by stabilization through solid particles present in the premix formulation [30], as well as the possible existence of mixed micellar structures consisting of LPC and PC in such systems [31]. Figure 12 depicts dried-up foam generated from Premix 5. It can clearly be seen that the phospholipids present in the premix form a particulate residue located where the lamellae of the foam were located. This indicates that solid particles from the premix primarily aggregate at the liquid–air interface, leading to more rigidity and further promoting foam stabilization [32,33,34].

## 4. Conclusions

The foamability of phospholipid mixtures is highly affected by the degree of hydrogenation of the compounds, as well as the presence of monoacylphosphatidylcholine.

Mixtures with a high fraction of LPC20 (hydrogenated PC + hydrogenated LPC), along with a non-hydrogenated phospholipid compound such as LPC90 or PL90G, especially resulted in fine, long-standing foams that showed almost no decay and only minute structural changes during the test period. In most cases, the mixtures that included hydrogenated PC (PL80H) were less favorable.

In the presence of an oil phase, these phospholipid premixes initially showed insufficient foaming behavior. The overall quality of the foam generated from Premix 5 plus MCT was substantially inferior compared to the pure premix, and Premix 3 was incapable of forming or stabilizing any foam at all.

However, the emulsions restored their foaming properties when LG was included as a cosurfactant. Contrarily, the use of LAPB as a cosurfactant resulted in more fragile foams with far coarser structures, indicating that LAPB is not suitable as a cosurfactant for these formulations.

The type of the used oil phase also had a substantial impact on the foam quality parameters. The use of medium- to high-polarity oil phases such as MCT and CO yielded the best results in terms of foamability and foam stability, while PO resulted in more inconsistent foaming behavior. In contrast, CO negatively affected the emulsion stability due to too-large droplets. The mean droplet size in the MCT or PO emulsions was significantly smaller, preventing creaming during a storage period of at least 3 months. Therefore, the medium-polar MCT was revealed to be an optimal oil phase, as it combines both good foaming properties and overall foam quality with sufficient emulsion stability, which neither the low-polarity PO or the high-polarity CO were capable of.

Pendant drop tensiometry and polarization microscopy suggest that the cosurfactants played the primary role in foam generation and stabilization in the investigated foamable water-in-oil emulsions, whereas the phospholipid blends primarily acted as an emulsion stabilizer in these formulations.

Finally, it can be concluded that foamable emulsions with satisfactory storage stability require both the phospholipid premix and a suitable cosurfactant.

## Figures and Tables

**Table 1:** Major constituents of the phospholipid compounds.

Constituent	LPC20	LPC90	PL80H	PL90G
Phosphatidylcholine, hydrogenated (hPC)	69.0%		80.9%	
Phosphatidylcholine (PC)		2.8%		96.1%
Lysophosphatidyl choline, hydrogenated (hLPC)	18.0%		2.0%	
Lysophosphatidyl choline (LPC)		89.3%		1.1%

**Table 2:** Composition of the investigated premix formulations.

Formulation	LPC	hLPC	PC	hPC
Premix 1	0.50% LPC90	-	0.50% PL90G	-
Premix 2	0.75% LPC90	-	-	0.25% PL80H
Premix 3	-	0.1875% LPC20	0.0625% PL90G	-
Premix 4	-	0.50% LPC20	-	0.50% PL80H
Premix 5	0.125% LPC90	0.375% LPC20	-	-

**Table 3:** Relative decrease in bubble count over time.

Formulation	BC(t_150_)/BC(t_0_)	BC(t_300_)/BC(t_0_)
Premix 1	36.25%	23.90%
Premix 2	39.78%	28.49%
Premix 3	72.21%	61.93%
Premix 4	37.26%	31.13%
Premix 5	62.29%	51.69%

**Table 4:** Compositions of the investigated emulsions.

Formulation	PL1	PL2	Cosurfactant	Oil Phase
**Premix 3**	0.1875% LPC20	0.0625% PL90G	-	-
Emulsion 1	0.16875% LPC20	0.05625% PL90G	0.45% LG	10.0% PO
Emulsion 2	0.16875% LPC20	0.05625% PL90G	0.45% LAPB	10.0% PO
Emulsion 3	0.16875% LPC20	0.05625% PL90G	0.45% LG	10.0% MCT
Emulsion 4	0.16875% LPC20	0.05625% PL90G	0.45% LAPB	10.0% MCT
Emulsion 5	0.16875% LPC20	0.05625% PL90G	0.45% LG	10.0% CO
Emulsion 6	0.16875% LPC20	0.05625% PL90G	0.45% LAPB	10.0% CO
**Premix 5**	0.375% LPC20	0.125% LPC90	-	-
Emulsion 7	0.3375% LPC20	0.1125% LPC90	0.45% LG	10.0% PO
Emulsion 8	0.3375% LPC20	0.1125% LPC90	0.45% LAPB	10.0% PO
Emulsion 9	0.3375% LPC20	0.1125% LPC90	0.45% LG	10.0% MCT
Emulsion 10	0.3375% LPC20	0.1125% LPC90	0.45% LAPB	10.0% MCT
Emulsion 11	0.3375% LPC20	0.1125% LPC90	0.45% LG	10.0% CO
Emulsion 12	0.3375% LPC20	0.1125% LPC90	0.45% LAPB	10.0% CO

**Table 5:** Comparison of the selected emulsions and the corresponding premixes.

Formulation	MBA(t_0_)	MBA(t_150_)	MBA(t_300_)	BC(t_0_)	BC(t_150_)	BC(t_300_)
**Premix 3**	6566.67	9125.00	10623.67	152.33	110.00	94.33
Emulsion 1	7371.67	14565.00	20089.33	138.33	70.00	50.33
Emulsion 3	7724.33	14242.67	23212.00	130.33	70.00	43.00
**Premix 5**	6347.00	10190.00	12268.33	157.33	98.00	81.33
Emulsion 9	7121.00	13203.00	20340.33	140.33	75.67	49.00

**Table 6:** Results of the droplet size determination (d_10_, d_50_ and d_90_ values) 24 h after manufacturing; mean ± SD, *n* = 3.

Formulation	d_10_ (µm)	d_50_ (µm)	d_90_ (µm)
**Premix 3**			
Emulsion 1	0.498	2.628	9.204
Emulsion 2	0.589	3.118	9.975
Emulsion 3	0.337	1.832	6.315
Emulsion 4	0.421	2.220	6.966
Emulsion 5	1.197	21.661	56.973
Emulsion 6	0.881	13.589	38.119
**Premix 5**			
Emulsion 7	0.638	3.517	12.443
Emulsion 8	0.562	2.870	9.807
Emulsion 9	0.434	2.327	7.556
Emulsion 10	0.405	2.214	7.341
Emulsion 11	1.292	23.846	68.532
Emulsion 12	1.206	23.016	59.856

## Data Availability

Not applicable.

## Supplementary Materials

The following supporting information can be downloaded at https://www.mdpi.com/article/10.3390/pharmaceutics14061212/s1: Figure S1. Foaming behavior of Premix 3 + 10.0% MCT. Figure S2. Premix 5 + 10.0% MCT at t_0_. Figure S3. Premix 5 + 10.0% MCT at t_150_. Figure S4. Premix 5 + 10.0% MCT at t_300_. Figure S5. Foaming behavior of Premix 5 + 10.0% MCT with 0.45% LG as a cosurfactant at t_0_. Figure S6. Foaming behavior of Premix 5 + 10.0% MCT with 0.45% LG as a cosurfactant at t_150_. Figure S7. Foaming behavior of Premix 5 + 10.0% MCT with 0.45% LG as a cosurfactant at t_300_.
