# Supplementary material for: Influence of Oil Polarity and Cosurfactants on the Foamability of Mono- and Diacylphosphatidylcholine Stabilized Emulsions
Source: Pharmaceutics. 2022 Jun 7;14(6):1212. doi: 10.3390/pharmaceutics14061212 (PMC9230088; doi:10.3390/pharmaceutics14061212)
Supplement: Supplementary file 1 [file pharmaceutics-14-01212-s001.zip › pharmaceutics-1705141-supplementary.pdf]

pharmaceutics

Supplementary Materials: Influence of oil polarity and cosurfactants on the foamability of mono- and di-acylphosphtidylcholine stabilized emulsions

Manuel Bunk and Rolf Daniels

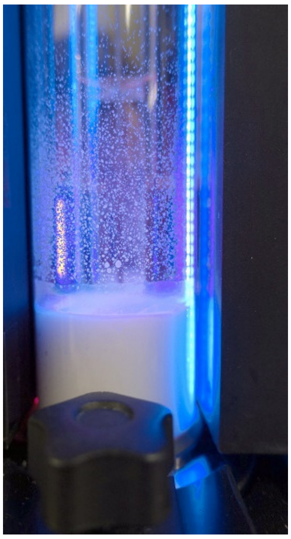

Figure S1. Foaming behavior of Premix 3 + 10.0 % MCT.

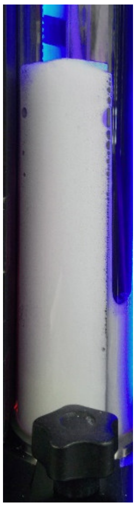

Figure S2. Premix 5 + 10.0 % MCT at t_0.

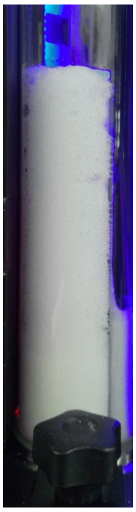

Figure S3. Premix 5 + 10.0 % MCT at t_{150}.

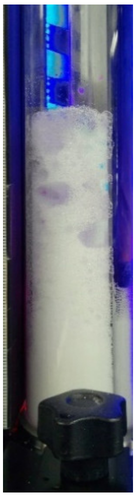

Figure S4. Premix 5 + 10.0 % MCT at t_{300}.

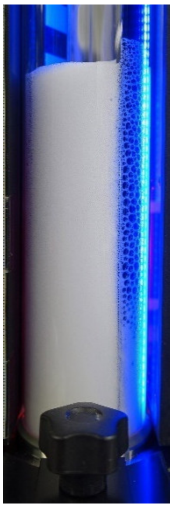

Figure S5. Foaming behavior of Premix 5 + 10.0 % MCT with 0.45 % LG as a cosurfactant at t_0.

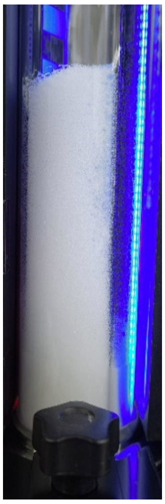

Figure S6. Foaming behavior of Premix 5 + 10.0 % MCT with 0.45 % LG as a cosurfactant at t_{150}.

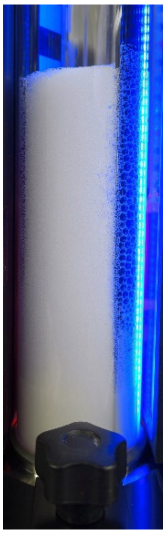

Figure S7. Foaming behavior of Premix 5 + 10.0 % MCT with 0.45 % LG as a cosurfactant at t_{300}.
